# *“Be explicit to be educated”*: using thematic analysis and co-design to investigate and understand sexuality education in Australian high schools

**DOI:** 10.1186/s12978-026-02288-y

**Published:** 2026-03-06

**Authors:** Ava Medley, Emmalee A. Ford, Shaila Dube, Sophie Maric, Sam Muller, Claire Taylor, Tania Day, Angela Dunford, Tanmay Bagade, Catherine Chojenta, Kirsty G. Pringle, Jessie M. Sutherland

**Affiliations:** 1 College of Health, Medicine and Wellbeing, The University of Newcastle, Callaghan, 2308 Australia; 2 Reproductive and Family Health Research Program, Hunter Medical Research Institute, New Lambton, 2305 Australia; 3 The Research Centre, Family Planning Australia, Newington, 2127 Australia; 4 Sexual and Reproductive Health Advisory Group, The University of Newcastle, Callaghan, Australia; 5 Maternity and Gynaecology, John Hunter Hospital, Newcastle, Australia

**Keywords:** Comprehensive sexuality education, Adolescents, Sexual and reproductive health

## Abstract

**Background:**

A comprehensive sexuality education is evidenced to provide youth with the skills and knowledge required to promote sexual and reproductive health. Comprehensive sexuality education is recommended globally as an effective intervention for a range of improved sexual health outcomes, including reducing sexually transmitted infections and teenage pregnancies. The Australian curriculum, despite attempts to provide comprehensive guidance for teaching, is unsuccessful in guiding adequate sexuality education for Australian adolescents; observably, its delivery is incohesive, with the relevance and scope varying among students. Simultaneously in Australia, we sustain alarmingly high rates of sexually transmitted infections in young people and an adult population with increasing uptake of assisted reproductive technologies. The diverse identities of adolescents and the changing nature of sexuality in society warrant an in-depth exploration into the ideas and issues of youth in order to provide sexuality education that is reflective of the population it serves and improve sexual and reproductive health outcomes nationally.

**Methods:**

Utilising thematic analysis methodology, nine focus groups consisting of 2–6 Australian participants aged 15–18-year-old were analysed to better understand what is required within sexuality education. For increased validity and interpretation of data, this project utilised a Youth Advisory Group of four members to validate the thematic analysis. The study’s Youth Advisory Group further engaged in the co-design and development of recommendations for delivering sexuality education in Australia that reflected the thematic findings from the focus groups and their own lived experience.

**Results:**

Thematic analysis revealed three prominent themes within focus group participants’ experiences; “culture of sex”, “content of sexuality education” and “sources of sexuality education beyond formal education”. The qualitative analysis revealed evidence of the complex relationship between these themes and Australia’s underperforming sexuality education. While these appear to be objectively undesirable and reflect the flaws within sexuality education, they presented guidance for the recommendations, informing the co-design conducted by the Youth Advisory Group.

**Conclusion:**

The findings of this study, together with relevant literature, provide evidence for the complexities of adolescent sexual experience and endorse comprehensive sexuality education in the context of contemporary Australian high schools, promoting the appraisal of current practice for improved sexual and reproductive outcomes in Australia, now and in the future.

## Abstract (plain language summary)

This study investigated how young Australians experience sexuality education in schools and what can be done to make it better. Sexuality education is important because it helps young people learn about sexual health, relationships, and how to protect themselves from things like sexually transmitted infections (STIs) and unplanned pregnancies.

Although Australia’s national curriculum tries to cover these topics, it often falls short. The quality, delivery and content of sex education seem to vary a lot depending on the school and the teacher, with many students feeling it does not meet their needs. At the same time, STIs remain common among young Australians, and more adults need help to have children through medical treatments.

To better understand what young people want and need from sex education, researchers talked with teens aged 15–18 in focus groups. A Youth Advisory Group also helped analyse the results and come up with practical suggestions based on both the focus group findings and their own experiences.

Three main issues came up: the way sex is viewed in society, what's actually taught in sex ed classes, and where young people learn about sex outside of school. These areas are deeply connected and highlight why Australia’s current approach isn’t working well.

The study provides valuable insights into how sex education can be improved in Australia by making it more relevant, inclusive, and reflective of young people's real experiences.

## Background

In adolescence, it is crucial that sexuality education is taught to promote and encourage positive sexual wellbeing with implications for future sexual health [1]. The internationally recognised best practice in sexuality education is Comprehensive Sexuality Education [2], outlined by the United Nations Educational, Scientific and Cultural Organisation (UNESCO) as a ‘curriculum-based process of teaching and learning about the cognitive, emotional, physical and social aspects of sexuality’ [3]. A defining feature of a comprehensive sexuality education is to foster an accepting, positive and respectful outlook on a range of topics that are inclusive to all, regardless of culture, religion, socio-economic factors, race, sexual orientation, or identity. This includes topics such as Relationships, Violence and Staying Safe, The Human Body and Development, Sexuality and Sexual Behaviour and Sexual and Reproductive Health. Content should be staggered to ensure that age-appropriate content is being delivered during the various stages of development, allowing knowledge to be built upon over the span of their education to increase both understanding and retention of information [3]. The delivery of sexuality education is also time-sensitive in the sense that it must be delivered prior to engagement in sexual activities for appropriate knowledge, attitudes and skills to be developed and implemented [4]. Globally, extensive evidence highlights a positive link between comprehensive sexuality education and improved sexual health outcomes. Such outcomes include decreased unintended pregnancies, increased condom usage, decreased sexually transmitted infections (STIs), an improved understanding of the diversity of sexuality, prevention of domestic violence, prevention of child sex abuse and reduced infertility risk behaviour [1, 5–8].

Current sexuality education delivery in Australia varies between schools [9]. The Australian curriculum stipulates that the responsibility for sexuality education implementation falls to state and territory governments [10], thus fostering the varying execution, adoption and level of enforcement of sexuality education. Furthermore, the ability of non-government schools to modify the curriculum and adopt the curriculum at their discretion exposes further potential for sexuality education to be modified in the education of some students [11–13]. The majority of Australian students attend government schools (63.4%), however a significant percentage (36.7%) of Australian adolescents attend Catholic or independent schools [14]. The interpretation of the curriculum by states with further adaptations at a school level, may be a significant factor in the inconsistent teachings of sexuality education and subsequent variation in learning outcomes among students [15].

These inconsistencies are reflected by the Australian adolescent population’s poor attitude and limited value they place on their sexuality education [16, 17]. National routine investigation into the range of topics covered in sexuality education identified that there is a lack of coverage of specific topics, including “safe sex between two men”, “safe sex between two women”, “anal sex” and “having sex with someone who has a disability”, suggesting the need for more inclusive topics and sexuality education “that is engaging, delivered more often, and covering a wide range of age-appropriate content” [16]. It is therefore prudent to investigate why sexuality education is not meeting the expectations of adolescents and how this evidence can inform potential solutions and subsequently improve the programs and their relevance. The implementation of contemporary sexuality education, with the opportunity for regular appraisal and refinement, would serve to mitigate concerns raised by the adolescent community regarding its relevance, particularly within a rapidly changing society.

The current implementation of sexuality education in Australia may be contributing to the persistently high incidence of sexually transmitted infections and suboptimal reproductive health outcomes at a national level. 69% of *Chlamydia* infections, 48% of *Gonorrhoea* and 31% of *Syphilis* infections are reported in those aged 15–29 years of age [18]. These STI notification rates increased by 17% for Chlamydia, 79% for Gonorrhoea and 95% for Syphilis between 2015–2019 [19]. Furthermore, The 7th National Survey of Secondary Students and Sexual Health identified that 60.6% of Year 10, 11 and 12 students reported that they were sexually active and of those who are sexually active, only 34.1% “always use a condom” [17]*.* In conjunction with these statistics, Australia’s high rates of subfertility are reflected in the large reliance on assisted reproductive technology (ART) for conception; 100,039 ART treatment cycles were initiated in Australia in 2022 [20]. Modifiable factors contributing to infertility include STI contraction, age, delayed childbearing, and lifestyle factors [21, 22]. Increasing fertility knowledge therefore has power to reduce preventable infertility through encouraging protective behaviours [21–23]. Despite both STI prevention and fertility education being recommended in comprehensive sexuality education, with significant existing evidence that the inclusion of these topics reduces these negative consequences [3, 4], the outcomes being achieved nationally suggest large gaps in STI prevention and awareness of reproductive issues [16, 17, 24].

The inclusion of youth within a community-academic partnership is known to improve the contextuality of the content delivered, optimising engagement, as programs are representative of the experiences of the target audience [2, 25, 26]. Without partnership of adolescents in the development of relevant educational programs, the level of participation and change in attitudes relies on the knowledge and assumptions of the Australian Curriculum, Assessment and Reporting Authority (ACARA) [27]. ACARA disclose consultation with “authorities, parent bodies, professional education associations, academics and business, industry and community groups” but fail to report any consultation with consumers; Australian students [27]. Specifically, sexual health delivery in schools is more successful when it encourages input from the learners [3, 4, 28]. Thus, within this study, we aimed to investigate the needs and wants of Australia’s adolescents within sexuality education to allow for better representation of and inclusion of youth issues. The study also serves to address the gross lack of consultation with youth for programs fit to educate them by working collaboratively with consumers to develop recommendations for school-based sexuality education. Ultimately, with improved understanding and consultation with Australian youth, national sexual health education would be optimised to prevent negative health outcomes and promote sexual and reproductive wellbeing.

## Methods

Ethical approval for the focus groups was obtained in the original survey [24], through the University of Newcastle’s Human Research Ethics Committee, under the protocol number H-2020–0018.

Participants were previously recruited in a study surveying reproductive and sexual health knowledge [24]. Participants were aged between 15–18 years old and attended or completed most of their secondary schooling in an Australian high school. This age group was selected under the assumption that Australian students should have been exposed to multiple sexuality education topics by this age, as per the Australian Curriculum [29], and are mature enough to reflect on this topic. At the survey’s completion [24], the 2,600 participants could self-nominate to participate in focus groups for further investigation, of which 196 participants self-selected their interest, and 24 were chosen to participate. Demographic data aligning with the Australian Bureau of Statistics sociodemographic factors was collected during the survey. Demographic data of the sample group can be found in Table 1. A total of 24 participants, over nine sessions (2–6 participants per session) engaged for up to 90 min with at least one researcher facilitating the sessions. The primary facilitator (EF) who conducted these focus sessions as part of an adjacent study did not complete the analysis in this study. The questions the facilitator asked aimed to explore the participants’ opinions about their sexual health education, knowledge, opinions and suggestions (Appendix 1). It remained a priority within these focus group sessions not to ask leading questions in order to collect unbiased responses.

**Table 1:** Demographic data of members of the focus groups. Total of 24 participants

Demographic category	Number (n)	Percentage (%)
Age		
15	6	25%
16	2	8%
17	11	46%
18	5	21%
State/Territory		
New South Wales	11	46%
Victoria	5	21%
South Australia	4	17%
Western Australia	2	8%
Queensland	1	4%
Northern Territory	1	4%
Identify as Aboriginal or Torres Strait Islander		
Yes	1	4%
No	23	96%
Born overseas		
Yes	5	21%
No	19	79%
Speak a language other than English at home		
Yes	4	17%
No	20	83%
Gender		
Man	3	13%
Woman	18	75%
Non-binary	2	8%
Agender	1	4%
Sexuality		
Heterosexual	9	38%
Bisexual	9	38%
Asexual	2	8%
Don’t know	3	13%
Prefer not to say	1	4%
Gender incongruent to sex at birth		
Yes	4	17%
No	20	83%

All transcripts obtained from the focus group sessions were de-identified and transcribed verbatim. The participants additionally had the opportunity to confirm, review or withdraw their contributions following a review of their session’s transcripts. These files were then uploaded to NVivo 14 software (Lumivero, Version 14) to complete the thematic analysis. Nvivo software was used to systematically organise and code the data, enabling the identification of themes and subthemes within the dataset [30]. The method of thematic analysis used was that of the Braun and Clarke *Reflexive Thematic Analysis Model.* To meet the analysis aims within this study, a constructionist epistemology was adopted, recognising the importance of recurring themes, but understanding this doesn’t equate salience within a theme [31]. Furthermore, following recommendations by the Reflexive Thematic Analysis model, an experimental orientation to data interpretation was used when ascribing the meaningfulness of responses given by participants, viewing responses as a direct reflection of the personal states held by the participant [32]. This reduced the likelihood of inferences to be made about the participants’ attitudes and opinions by choosing to ignore the potential social context and construct systems of meaning which offer interpretations rather than explicit communications by the participant.

### Study’s youth advisory group

Ethical approval for the focus groups was obtained in the original survey through the University of Newcastle’s Human Research Ethics Committee, under the protocol number H-2020–0301.

Members of the Youth Advisory Group were voluntarily recruited via word-of-mouth and physical flyers. The eligibility criteria included that participants were between 18–23 years old and were comfortable discussing issues pertaining to sexuality and reproduction. Additionally, participants had to have completed the majority of their schooling in Australia. Demographic data of the study’s advisory group was recorded in a survey (Table 2). Each meeting with the advisory group was held virtually and was approximately one hour long, conducted on a monthly basis. The members were remunerated $40 AUD per hour for their time in line with NSW Health consumer remuneration guidelines [33]. Prior to each meeting, a meeting agenda was disseminated for members to begin formulating ideas for discussion. The session agenda was dependent on the stage of research the project had achieved but was significantly guided by the desires of members and what they felt was an important topic for discussion. The advisory group members were considered co-researchers; their input was deemed as valuable as any other researcher, and their own individual lived experience recognises them as specifically informed about the topic.

**Table 2:** Demographic data of members of the youth advisory group. Total of 4 participants

Demographic category	Number (n)
Age	
20	1
21	0
22	2
23	1
State/Territory	
New South Wales	3
Australia Capital Territory	1
Identify as Aboriginal or Torres Strait Islander	
Yes	0
No	4
Born overseas	
Yes	2
No	2
Speak a language other than English at home	
Yes	2
No	2
Gender	
Woman	3
Non-binary	1
Sexuality	
Heterosexual	3
Asexual biromantic	1
Gender incongruent to sex assigned at birth	
Yes	1
No	3

**Fig. 1:**
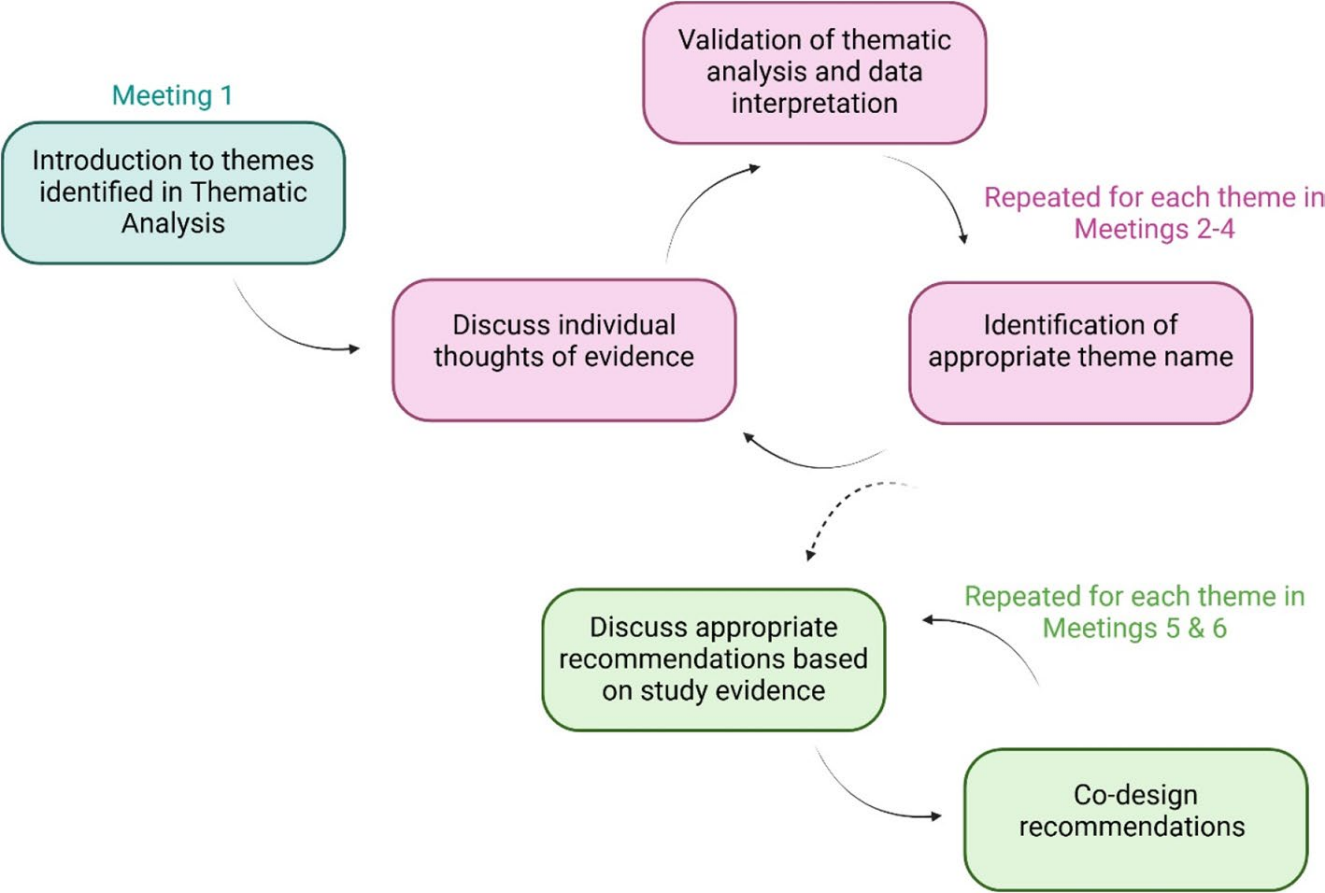
Co-design process utilised with the youth advisory group when developing recommendations

Through the methodology of co-design [34], an iterative cycle of reflection, data collection and action was enacted as shown in Fig. 1. The cyclic methodology allowed for rigorous representative research to be conducted that was better informed by consumers. Every member attended each of the six meetings, and the sessions ranged from one to two hours in length. The group discussions on issues, suggestions and recommendations were all guided by the members’ contributions and priorities. The first meeting was centred on member introductions, establishing an understanding of the study’s goals, and building rapport. In subsequent sessions, the facilitator guided the iterative nature of the methodology but refrained from guiding the opinions and suggestions made by members. At the commencement of each session, participants were provided with the meeting minutes and meeting recordings to allow for reflections on the outcomes and recommendations in the final meetings.

The recommendations made for Australian high schools regarding the delivery and content of their sexual and reproductive education were informed by the thematic analysis conducted in this project in conjunction with the Youth Advisory Group. Each recommendation was contextualised by thematic analysis data, alongside recommendations by the *International Technical Guidance for Comprehensive Sexuality Education *[3]. These recommendations were discussed as part of the meetings; the key theme(s) being discussed, paired with the corresponding *Key Concept* (detailed in the *International Technical Guidance for Comprehensive Sexuality Education *[3]). Prior to the meeting, members were asked to review the UNESCO guidance and outcomes outlined in previous meeting minutes, with the opportunity to submit initial suggestions for recommendations via a poll open field response. This allowed members to give suggestions independent of each other in the meeting to minimise confirmation bias among members. In addition to the existing guidance by UNESCO for sexuality education, the recommendations developed in this research project are specific to the Australian youth population, with clear appraisal for the needs and preferences of Australian youth. For accuracy, two clinicians (AD and TD) who practice reproductive medicine, reviewed the proposed recommendations to ensure the terminology was appropriate and clinically relevant.

## Results

Regardless of the diverse demographics and individual experiences of focus group participants, universal themes became apparent across the data set. Three themes and eight subthemes were identified. The three themes were: Culture of sex, Content of sexuality education, and Sources of sexual health information beyond formal education. See Table 4 in Appendix 2 for evidence of these themes.

### Theme 1: Culture of sex

#### Subtheme 1.1: Sex being taboo

Participants recognised that there is a societal perception of any conversation about sex to be taboo, stating their parents, teachers and online community are inclined to avoid conversations about sex. They additionally introduced the notion that they “*copy*” their superiors in what is believed to be acceptable within society, perpetuating the practice of ignoring such issues because they are led to believe it is something that we “*should not talk about*”.

*I think we copy like adults and what they see is like acceptable and what they think is like okay to talk about and so like from that young age you see like your teachers don’t want to talk about it. Your parents don’t want to talk about it, people on the internet that you found on YouTube are a little bit awkward about talking about it too. Um it kind of just gives this idea that like it’s not something that you should talk about* (Female, 17, Bisexual)

The shame around discussing sex broadly was also relevant when discussing health issues resulting from sex, such as sexually transmitted infections.

*Infections and diseases they’re definitely like this massive taboo like if you have one just don’t say anything, and don’t talk about them and they don’t exist* (Female, 17, Bisexual)

#### Subtheme 1.2: General poor sex culture

Participants raised issues about the lack of consideration for the consequences of sex, particularly by their male peers engaging in heteronormative sex. Among males, the gratification from others for engaging in sexual acts was discussed as being more important than the potential consequences that eventuate from sex. However, the same sexual acts and behaviours of females were detested and substantiated grounds for ‘slut-shaming’ despite the individual being subject to the same peer pressure.

*So I’m gonna have sex, don’t care about the consequences because in the end, If I knock someone up I can leave. That that’s their mentality. It’s a lot of peer pressure, it’s kind of that societal expectation to, again, guy sleeps with girl, guy gets, you know, a high five from his friends. Girls sleeps with guy, girl gets slut-shamed* (Female, 17)

This discrepancy in how different genders are viewed differently for the same behaviours was not only observed within focus group discussions, but was identified as a wider ideology that youth are keenly aware of. This phenomenon was epitomised by a male participant who used the term “*misogynistic*” to describe fellow male classmates.

*As boys we have a tendency to have, to be honest, a sort of misogynistic, very old, poor, I don’t know, behaviour* (Male, 15, Heterosexual)

### Theme 2: Content of sexuality education

#### Subtheme 2.1: Motives of sex for youth

Youth reported that the understanding that youth are unlikely to be engaging in sexual acts for reproduction was not addressed in sexuality education. Participants expressed a desire for content angled towards the various contexts (and motivations) for engaging in sex through multiple expressions of curiosity towards sex for pleasure and intimacy, rather than for procreation.

*But I also think sex education really only teaches people what having sex is not how to have sex, because I feel like really just saying like a penis can go in a vagina that’s, that’s not the only way to have sex and also how to have pleasurable sex like not for reproduction. I feel like it’s something that’s not talked about, which people who are younger, are probably not doing it for reproduction* (Nonbinary, 17, preferred not to state sexuality,)

*The whole idea of sex in terms of sex ed is for procreation rather than anyone’s pleasure and being intimate with a partner, and the positives of that can generate, that idea—definitely not touched on* (Female, 17, Bisexual)

#### Subtheme 2.2: Lack of fertility content

When reviewing focus group transcripts, it was evident that the convention in which school-based sexuality education addresses fertility falls short of participant expectations. Fertility content, or lack thereof, was described as “*never*” being taught to participants in their high school sexuality education, prioritising fearmongering to encourage abstinence.

*I have never been taught anything to do with fertility, because if they are to teach anything in sex ed it’s more just trying to scare us into not doing it since it is basically not teaching us anything about fertility because that will kind of like, they think it’s encouraging us* (Female, 15, Bisexual)

With anticipation of their future parenting intentions, participants admitted to being concerned about infertility when progressing into adulthood.

*Yeah, we never touched on infertility at all or anything in regards to that which is something that I worry about personally like going into like my adult life* (Female, 17, Bisexual)

#### Subtheme 2.3: Lack of LGBTQIA + inclusive content

Inclusive LGBTQIA + content was also overlooked in sexuality education of participants. Specifically, participants expressed curiosity about non-heteronormative forms of sexual interactions. However, this curiosity was often seemingly met with teachers who were either not knowledgeable in such topics or were reluctant to address them.

*Our male teacher didn’t know pretty much anything about the female system or how to have like sex with it and so when I asked how two women could do it or how two men, he had no idea* (Nonbinary, 17)

This approach to sexuality education, where female-with-male sexual interaction is exclusively taught, not only fails to address alternative mechanics of sex but additionally denies crucial safe sex information to students in non-heteronormative relationships.

*We basically just got taught the examples of sex basically we only got taught just you know just, male and female. And then contraception just, yeah just, you got a male, female, like, um, yeah I didn’t know about that whole, you know, how you’d have to use it for two men* (Male, 18, Heterosexual)

#### Subtheme 2.4: Sexually transmitted infection content

Participants reported semantic understanding of sexually transmitted infections through their sexuality education but recognised a lack of deeper comprehension of the topic. Interestingly, participants repeatedly used rhetorical language, suggesting attempts to draw attention to their lack of understanding.

*I know what all the STIs are? Goodness no, like, do I understand them all? No, of course not. And the fact that they spread over so many, but didn’t go into them in as much detail* (Female, 16, Bisexual)

This disconnect is further affirmed through the quote and question, by another participant. The manner in which participants articulate their experiences regarding this aspect of sexuality education is consistently framed in a way that conveys their lack of comprehension, appearing to convince the researcher of their superficial grasp of the content.

*Chlamydia. of course, I know what I know what the word is, I don’t really know what it is. I know it’s an STI. Right?* (Female, 17, did not yet know sexuality)

#### Subtheme 2.5: Vague and inexplicit content

The vague nature of sexuality education persisted across key learning areas, with content being taught superficially and without clear purpose. Multiple participant recounts described the method of teaching consent to be inadequate whereby schools utilised an analogy of offering someone a cup of tea when educating about sexual consent.

*I’m sure you’ve heard of it, the tea video for consent. And I feel like it’s kind of it’s kind of backwards, it defeats the purpose in my opinion because we have to be explicit to be educated.* (Female, 16, Heterosexual)

The maturity of participants was reflected through the recognition that using analogies of “*forcing tea down someone’s throat*” for something that has serious consequences is inappropriate and reduces the severity of an act of sexual assault. Generally, participants expressed a dissatisfaction with the avoidance of explicit discussion around sexuality education topics and their frustration with the use of far-stretched metaphors.

*The tea video like people don’t want an analogy of like don’t force it down to someone’s throat. No don’t force somebody to have sex if they don’t want to wait. We’re like unintentionally glossing things over and in that sense we’re lessening the idea of the consequence, like, okay, forcing tea down someone’s throat you’re going to burn your throat but or forcing someone to have sex you could give them an STI. You couldn’t leave them with permanent trauma, you can leave them with a child.* (Female, 17)

### Theme 3: Sources of sexual health information beyond formal education

#### Subtheme 3.1: Impact of digital media on sexuality understanding

Participants discussed the plethora of media platforms accessible to youth, which may glorify sex and sexual issues. Consequences of such consumption, whereby youth potentially idolise content, was spoken about with particular concern regarding the romanticisation of sexual assault and abuse in an environment that doesn’t “*have adequate consent education within schools*”.

*So I think a lot of the media like that, whether it be made movies, TV, pornography, whatever. A lot of the time they glorify and romanticise borderline assault and abuse and when we don’t have adequate consent education within schools already, we can’t see that when we’re consuming it we consume it and idolise it* (Female, 17)

The reality of the media being where youth consume sexual content further highlights the potential for unhealthy expectations for sexual interactions.

*Obviously the media is a big portrayal even in movies that’s where you first come across a lot of like interactions. And then the idea of consent is obviously not the core of a lot of these movies* (Female, 17, Bisexual)

*And then people get very misinformed ideas when they consuming pornography and the way that relationships should work in terms of that I’ve seen people’s ideas and things like that, and how skewed they are from reality type of thing, because of that, just not being able to separate that and, like, that’s fiction, from like the real world type of thing* (Female, 17, Bisexual)

#### Subtheme 3.2: Sexual health misinformation

When accessing sources of sexuality education, the legitimacy and accuracy of the source was questioned by participants. Uncertainty is introduced when accessing sexual and reproductive health information online, established when participants require additional information, but are met with the chaotic counsel of the internet.

*Yeah, and the internet is also a very dangerous place in terms of talking about sexual stuff, because you don’t know who the age of the person is who you’re talking to, the validity of the article you’re looking at. Even if the source, you have is serious or satire, you never know, like, it’s a complete grey area* (Agender, 17, Bisexual)

### Recommendations for sexuality education

Recommendations for Australian sexuality education were co-designed with the Youth Advisory Group (Table 3). The recommendations were made in consideration of the results from the Thematic Analysis, each member’s lived experience as a young person experiencing Australia’s sexuality education and elements from UNESCO’s International Technical Guidance for Sexuality Education [3]. Additionally, these recommendations were reviewed by reproductive clinicians to ensure accuracy and clinical relevance. Suggestions for implementation were later added (following meetings with the Youth Advisory Group) to suggest an appropriate level of intervention and how the recommendation might be applied.

**Table 3:** Co-designed recommendations made with the Youth Advisory Group for delivering sexuality education in the Australian context

Related theme	Recommendation	Key suggestions for improvement	Suggestions for implementation
General Recommendations	Consistent, comprehensive sexuality education in schools	Address all UNESCO key learning concepts developmentally, with clear goals and outcomes. Utilise learning objectives that are logically staged and reinforced at each age group (e.g. ages 5–8, ages 9–12, ages 12–15 and ages 15–18 +), with concepts for younger students typically including more basic information, less advanced cognitive tasks, and less complex activities • For example, in ages 5–8, discussing that everyone has the right to decide who can touch their body; in ages 15–18, after progressively learning about consent, discussing the factors that can impact the ability to acknowledge and to give consent	States to consult UNESCO *International technical guidance on sexuality education *[3] to inform their state curriculum
			States to utilise a tool such as the *Sexuality Education Review and Assessment Tool (SERAT) *[35] when reforming sexuality education curricula
			States to endorse this curriculum within their state’s governmental and non-governmental schools
	Encourage learner-centred approaches	Promote the opportunity to collaborate with students to help determine the students' needs and behaviours	Schools to encourage the involvement of students in their sexuality education and support their needs
			For example, encouraging school leaders or student groups to co-design and evaluate sexuality education lessons
		Create platforms and safe spaces within schools for students to ask questions without fear of judgement or misinformation	Schools to seek out and address the needs of students in their sexuality education
			For example, including a question box in sexuality education lessons, where students can anonymously ask questions and have them answered
	Professional development training for teachers and pre-service teachers	Support teachers to plan and implement sexuality education initiatives. Promote training programs for educators to ensure they have access to up-to-date resources and information to effectively and confidently teach sexuality education	National and state governments to increase resources for the appropriate training of teachers to educate on sexuality education
			Universities to consider the value of explicit training in sexuality education for their pre-service teachers. *National Teacher Preparation Standards for Sexuality Education* [36] provides an example of guidance for higher education institutions
Culture of Sex	Discuss content within a variety of different contexts that influence sexual experience	Address how societal and gender norms, peer pressure, and expectations may positively or negatively impact individual attitudes and behaviour. Consider how the content of sexuality education is influenced by students’ contexts, to alter the prioritisation of specific learning concepts	Schools to recognise the environmental and cultural nuances in teaching school-based sexuality education
			Schools to adopt school-specific policies to suit the contexts and needs of their students. This is detailed in pages 86–87 of UNESCO’s *International technical guidance on sexuality education *[3]
			For example, students may be using shameful language around being a “virgin”, and so lessons may have a specific focus towards peer pressure, discussing the current negative culture at the school
Sources of Sexuality Education beyond Formal Education	Utilise real-world scenarios and support systems to educate on sexuality comprehension	Educate students on the fictional portrayals of sexuality (including relationships, fertility, consent, STIs) within mass media and society, and how this is potentially harmful and misleading	States to ensure curriculum develops critical thinking skills in the context of external sources of sexuality education. Examples of this content can be found in UNESCO’s *International technical guidance on sexuality education *[3]
		Develop critical thinking skills on how to evaluate the credibility and reliability of media sources providing sexual and reproductive health information	
		Promote active involvement and support for parents, guardians, and community members in their children's sexuality education by providing resources, guidance, and opportunities for engagement	Schools to encourage community involvement in the implementation of sexuality education content. Examples of this can be found in pages 87–88 of UNESCO’s *International technical guidance on sexuality education *[3]
Content of Sexuality Education	Better utilisation of UNESCO guidelines with consideration for individual student needs and desires for content	Implement direct scenarios and explanations to describe sexual acts and consent, replacing vague language and euphemisms, to reduce uncertainty and convey consequences of non-consensual sex (e.g. trauma, STIs and unintended pregnancy) and the legal repercussions of such acts	States to align curriculum to UNESCO’s *International technical guidance on sexuality education *[3]
		Explore current research regarding adolescent sexual behaviour, emphasising that many young people engage in sexual activities for a variety of reasons (e.g. pleasure, intimacy and self-exploration) other than for reproduction • Incorporate and explore all genders and sexualities when teaching sexual education content to expand understanding of LGBTQIA + and increase queer visibility across all issues and topic areas ◦ For example, use inclusive, non-gender specific terms when describing sexual intercourse, pleasure, contraception, sexual partners and consent	State government to endorse the use of curriculum in government and non-governmental schools
		Provide comprehensive education on all STIs, inclusive of all identities. Discuss prevalence, prevention and treatment relevant to current Australian statistics	
		Provide comprehensive education on reproduction and fertility, and the role of contraceptives and their effective use. Consider factors that affect fertility in all genders and the limitations of reproduction. Discuss prevalence, prevention and treatment of reproductive issues and pathologies to include tubal and unexplained infertility, polycystic ovarian syndrome, PCOS, and endometriosis	

## Discussion

In this study, a thematic analysis of focus group transcripts of Australian high school students was conducted to explore ideas and issues within their sexuality education. Our analysis revealed three prominent themes: “culture of sex”, “content of sexuality education” and “sources of sexuality education beyond formal education”. These themes maintain a dynamic relationship with one another, each independently influencing an adolescent’s individual sexuality education experience and their understanding, illustrating evidence of a larger issue within society. Through the identification of this thematic data, informed, context-relevant recommendations developed with a Youth Advisory Group emerged. The following discussion will attempt to explore these themes in parallel and as a fluid issue to understand the complexity of sexuality education in Australia, substantiating recommendations made.

This study identified a pivotal discourse regarding the culture of sex, underpinned by sex as a taboo topic, modelled by those in positions of power. Despite the introduction of basic sexual health education in Australia prior to the Second World War in response to the perceived threat of sexually transmitted diseases, it was masked in conservative values [37], giving support to the idea that ‘secrecy and indecency are necessary associates of sex’ [37]. Despite now being able to reflect on such naivety towards sexuality education, the results from this study suggest this outlook prevails in K-12 educational institutions today. This sentiment has been identified in other Australian adolescent studies where students discuss that sexuality education is taught, refraining from discussing sex with the lens that it is a topic to be shied away from [38]. It appears the need for improvement of sex education is influenced by a society in which conversations about sex are still being avoided and adolescents are considered to need “protecting” from the realities of sex, maintaining this cultural norm of sexual ignorance being equated with sexual innocence [39]. This emphasis on “safety” and protecting adolescents from the risk of sex [40], is clear within the sexuality education experience of this study’s participants. This study highlights the asymmetry and irony within this idea, whereby those who would naturally be considered immature, young people, are more inclined to seek out understanding about a mature subject than the mature community themselves. By youth challenging the current culture of ignorance and taboo regarding sexuality and reproductive health, they demonstrate a desire to resist this societal convention. It is only through shifting the lens of sex to be something normal and developmental that we can begin to break down this enduring negative attitude towards sexuality education [41].

Within this study, the justification for engaging in sex due to peer pressure, and the use of normalisation of sex to motivate others to also engage in sexual activities, was observed. Both the perceived and experienced societal expectations to engage in sexual acts was sustained by youth, situating this study’s findings into the larger paradigm of Australia’s culture of sex [42, 43]. Within the literature, peer pressure has been identified as the “social influence of peers on an individual to conform to a particular way of acting or thinking” [44]. Youth within this study contribute further evidence to this hypothesis; peers encouraging the dismissal of personal values to participate in the culturally accepted sexual behaviours other peers engage in. This practice was particularly strong within males engaging in heteronormative sex, contributing to normalisation of attitudes of misogynistic nature. Interestingly, misogyny was another key discourse within Australia’s enduring negative sex culture [45] that the youth in this study identified. Most noticeably, males within this study admitted understanding the existence of this rudimentary culture, yet expressed no inclination to change their views, emphasising the power of Australia’s culture in modelling the norms adopted by our youth. This study’s recommendations sought to consider the impact of this culture on sexuality education, and consequently, multiple recommendations were developed to provide a more balanced view on sex liberated from misogyny and gender expectations.

For a cultural shift to occur, it is necessary for youth to engage in their sexual and reproductive health education to develop the knowledge and skills of this educational content. This study revealed that this is dependent on both the ability of youth to relate to the content being taught, and whether the content captures a comprehensive range of issues. The disconnect between one’s own sexual endeavours and what is being taught within sexuality education appears to be due partly to the inability of participants to associate with content that does not relate to the sexual practices and opinions held by youth themselves. There was a clear consensus that youth are not engaged in sex for reproduction, instead they engage for pleasure, yet their education disregards this motivation. The recommendation made with the advisory group, naturally, was to include these various motivations for sex. This study’s findings align with international discourse of inclusion of pleasure for all individuals within sexuality education [38, 46, 47].

The irrelevance youth perceive in their sexuality education is made worse through disparities and inadequacy in fertility education. Knowledge of fertility issues and content remains low in Australian adolescents [24], which, paired with this study reporting on the lack of fertility content within the Australian curriculum, is unsurprising [48]. Logically this has led to a strong emphasis on improving fertility education with Australia [49, 50]. Yet it seems the Australian curriculum remains rooted in values of pregnancy prevention, with a clear priority to reduce teen pregnancy rates atop reproductive health [24]. However paramount as reducing teen pregnancy may be, within the context of Australian teens this issue is of minor significance when adjacent to the prevalence of teenage mothers which has more than halved since 2011, representing only 1.5% of all mothers [51]. It could be concluded that the conservative values of Australia’s past [37], as previously discussed, hold true with intimidation tactics that are overly concerned with “protecting” adolescents from the realities of sex. The prominent subtheme, lack of fertility content, that arose in the thematic analysis could be attributed to the original survey’s [24] purpose to investigate fertility knowledge and thus influence the topic of fertility arising within focus groups. However, while the researcher introduced the topic, it did not shape or influence the nature of the experiences shared by the participants. Although the theme’s salience within focus group session’s may yet still prompt concerns of bias, its validation by the Youth Advisory Group reinforces its recognition that fertility is a significant issue in Australia’s sexuality education.

Inclusive sexuality education has long been sought after in Australia and this aspiration was voiced within this study, contributing to the consensus that Australia’s sexuality education is not considered inclusive of diverse genders and sexualities. Alongside the extensive dialogue and petition by the focus group participants, the study’s Youth Advisory Group felt it appropriate to explicitly incorporate inclusive language and LGBTQIA + recommendations made to Australian high schools. With 11% of Australians under the age of 25 identifying as LGBTQIA +, this is expected [52]. However, the proclivity of current sexuality education to focus on solely reproductive, heteronormative, penetrative sex, over-exemplifies one instance of sexual activity and fails to address the plethora of sexual experiences and activities of a wide range of individuals beyond cis men and women [53]. This reluctance for inclusive content discussed by participants, furthering unfamiliarity with key contraceptive, disease prevention and consent information for healthy relationships, is consistent among other studies of the LGBTQIA + community [54, 55]. Grant et al. highlighted experiences in the sexuality education of Australian queer women vastly similar to those within our study, disclosing they too, believed information they received was not relevant to them due to the heterosexual focus of content [54]. For those in non-heteronormative relationships this contributes to inequitable opportunity for sexual health. The desire for explicit inclusive LGBTQIA + content has repeatedly been met with dispute from religious and political groups concerned with content to be “undermining” traditional gender roles [56]. As the curriculum reflects Australian political and social beliefs [57], either our society as a whole does not value inclusivity, which the study’s participants reject through focus group discussions, or the curriculum remains overly concerned with pacifying opposing educational values through ambiguity. The political convenience in remaining enigmatic shifts the onus to schools and teachers to tailor sexuality education to a diverse group of students [57]. Educators dictating as to whether this occurs, based upon their own motivation, leaves many adolescents without appropriate sexuality education, as was discerned within this study. It is therefore pivotal a broader scope of gender and sexuality is incorporated throughout all topics of sexuality content; this belief echoed by our Advisory Group’s recommendations.

In contrast to the minimal or lack of coverage in fertility and LGBTQIA + topics, for STI content, participants were primarily concerned with the depth and transparency in their education. This sentiment was shared among other Australian students, who reported that STI content was taught superficially [9, 58]. Up-to-date, comprehensive sexually transmitted infection education was thus another direct recommendation that emerged from this study. The large number of young people being diagnosed with preventable sexually transmitted diseases emphasises a clear lack of understanding of the transmission and awareness of STIs. In 2022, Australian 15–29-year-olds represented 69% of Chlamydia cases, 48% of Gonorrhoea cases and 31% of Syphilis cases [59]. The overarching sexual culture and behaviours within Australia, and the direct hesitancy for a sex-positive education to be taught to Australia’s adolescents, have been established as problematic within this study. It is not presumptuous to assume that this lack of depth within STI content is a by-product of the taboo Australia holds for adolescent sex, even more so when participant verbatim supports this theory. The stigmatisation of sexually transmitted infections is a perpetual issue when poor sex culture is not addressed. To break this cyclic relationship, STI content should be addressed appropriately to reduce the ever-present taboo we observed in this study, as recommended. Complementing this, without a broad range of contexts and scenarios considered when educating on sexually transmitted infections, a level of uncertainty regarding who the content is relevant to remains, particularly in non-heteronormative relationships [58]. Independently, poor STI education manifests in broad, universal STI rates in adolescents. However, when paired alongside shortfalls in LGBTQIA + content, it could explain why gay and bisexual men are disproportionately overrepresented in STI rates [59]. The inclusion of LGBTQIA + STI content presents a potential opportunity for both the depth of this content to be strengthened and the previous issue of diversity within sexuality education to be better addressed.

Participants discussing the inclusion of consent education within sexuality education lessons were expected given the Australian Government mandating consent education in schools in 2022 [60]. Assuming educators are adhering to this new legislation and educate on power, coercion and sexual consent within these lessons as instructed, we hypothesise that this accounts for the lack of conversation of gender and power within focus group sessions, despite this topic remaining crucial within CSE [61]. The manner in which consent is being taught in Australia, appears however, surprising. Woodley et al., a study engaging with Australia’s adolescents, found that consent education now appears to dominate sexuality education, with neglect for other topics [62]. Our work both contrasts and compliments findings from Woodley et al., our participants not relaying the idea of consent “dominating” lessons, but agreeing that consent was included within lessons, albeit poorly. This study’s findings indicate that consent education was not taught directly or with explicit language but rather with the use of analogies. For example, the use of “the tea video” [63] was discussed as a vessel for educating adolescents on consent education in this study. It is established that the use of analogies is not uncommon in education and can be a useful tool in increasing comprehension through well-constructed comparisons [64]. However, in the context of sexual health education, the juxtaposition of using the analogy of forcing someone to drink a cup of tea to explain sexual assault is not a well-constructed nor appropriate comparison. Optimistically, the tea video does allow for and encourage more nuanced discussion within the classroom and is potentially a great tool to introduce the concept of consent [65]. Nevertheless, Australian youth in this study find the context in which this video has been used, either independently or in an environment where there may be varying levels of understanding of consent, to be inadequate. Hence, a clear recommendation that specifically encouraged the dismissal of “vague language and euphemisms” within school-based sexuality education was endorsed by the Youth Advisory Group.

Given this study’s position alongside the extensive literature, it is justified to affirm Australia’s sexuality education as insufficient according to youth. As this study further explored, an educational system that does not satisfy the standard for sexuality education, whether this be due to its lack of relevance or comprehensiveness, leaves adolescents to rely on alternate forms of information to source sexuality education. The influential potential of these informal sources of information on sex and sexuality understanding was regarded as substantive and contributed to young people being misinformed about sex and relationships, and to adopt false perceptions of real-life sexual experiences. The unrealistic portrayal of sex and relationships in the media admittedly concerns youth, with worries that widely idolised stereotypes will continue, in alignment with existing literature [66]. The ability of adolescents to consume unhealthy portrayals of sex and relationships without a critically informed consciousness was brought forth given the familiar propensity for media to make sexual innuendos and imply sexual meaning. Whether consciously or subconsciously, it is understood that young adults will consume media as they progress to adulthood, and that this can have a key influence on their sexual socialisation [67]. Generalisations made by participants regarding permissive sexual attitudes and the influence of media on their peers’ experiences align with published literature that positively associates social media use with these attitudes [68]. This study did not attribute these unrealistic expectations with conscious media consumption, but rather highlighted the risks associated with the limited sexuality education and exposure to such content.

Beyond media having the potential to influence adolescent ideas and attitudes, this study found other external sources of sexuality education to misinform youth. The current need to assess whether a source is accurate has been identified within Australian youth previously, which reported young people being required to distinguish factual sexual health knowledge from opinion or experience [69]. It appears a shared experience among Australian youth that accessing sexual health information online becomes a process of reliability assessment [70]. The promotion of critical thinking skills in the context of sexuality education in media was, for this reason, recommended within this study. Despite this uncertainty in youth when accessing sources of sexuality education, there is obvious potential for the internet to offer sexual and reproductive health information with its ease of access [71]. Thus, to improve the ability of young people’s understanding of what an appropriate source of information is, would reduce the propagation of inaccurate ideas and false “facts” about sexuality and offer opportunity to strengthen one’s ability to critically consume sexuality information. Considering the inequities of access and exposure to appropriate sexuality content in school settings identified in this study, resources outside of formal education become essential to some youth when exploring this content. The capacity of such accessible sources to inform youth could complement school-based education to strengthen understanding or alternatively provide a safe source of information for those in sheltered settings.

### Study limitations

The use of focus group data that was collected by another researcher may have introduced the opportunity for bias. Researchers can introduce their own interpretation bias when collecting and analysing data; thus, two sets of researcher bias may have influenced the data set used: one during data collection and one during analysis. However, due to the focus groups being conducted prior to this study, it consequently reduced the likelihood of leading questions relative to this study’s outcomes to be asked as this study’s research question was not considered. This is not the case for the subtheme of fertility-related content that arose, as this discussion point could be attributed the original study’s research question. Nevertheless, the use of an advisory group within this study that validated thematic analysis significantly reduced this bias.

It is a limitation when using focus groups that the participants may not represent an accurate diversity of the population. The focus group participants within this study were reflective of the broader Australian population in terms of sexuality, age, language spoken at home, Aboriginal and Torres Strait Islander status and location. However, the focus group participant population did not reflect the gender proportions within the Australian population. The inclusion of only four male participants to contribute to the thematic analysis reduces the representation of male ideas, and values. Additionally, the number of participants in the focus group population may limit the ideas and experiences that are collected. Sample bias increases the likelihood for incorrect assumptions to be concluded and disrupt a study’s validity [72]. This may contribute to the saturation of ideas however, the introduction of the Youth Advisory Group, a separate subgroup of Australian youth, reduces the likelihood for sample group biases as they independently validated the ideas of the focus groups. Furthermore, is important to note the diversity of ideas and issues that become present with the use of a focus group. Despite all participant voices being valued as equal, within the scope of this study, it becomes inefficient to try and discuss every idea suggested. It was thus a key role within the Youth Advisory Group to prioritise the themes and ideas discussed within the focus groups.

A further limitation of this study was the lack of diverse demographic representation of those who engaged in the co-design process; in this study, the Youth Advisory Group. As the Group had a total of four members, an accurate representation of the Australian youth population could not be achieved without the recruitment of additional members, highlighting recruitment challenges and consequent limitations. Quantitatively, an optimal balance of the number of members is unknown for a study such as this; our Advisory Group may have benefited from a broader range of experiences with additional members however, excessive consultation may have inhibited the cohesive meetings and the recommendations made [73] When working with a Youth Advisory Group it is key to have meaningful engagement in the development process with the ability to build rapport with the research team. Given the smaller-sized Advisory group within this study, values of teamwork, trust and collaboration were able to be prioritised as significant time was allocated to supporting group dynamics for purposeful participation [74]. Nevertheless, qualitatively, we did not have any male participants to provide guidance and contribute to the recommendations for Australian high schools when delivering sexuality education. This is a clear limitation of our Advisory Group and may have reduced the discourse of issues discussed from a male perspective. Specifically, in discussions regarding recommendations to address peer pressure between sexes, the male perspective could have strengthened or reformed this guidance. Our Youth Advisory Group furthermore, did not have a member who identified as Aboriginal and/or Torres Strait Islander. Those who are part of this community have a specific set of needs and desires within Australia’s sexuality education [70]. Although our focus group participants were diverse with Aboriginal participants, and as a group mirrored many of the perspectives and experiences of Aboriginal and/or Torres Strait Islander students in Australia’s sexuality education [70], it is a clear limitation that we did not have representation of Aboriginal and/or Torres Strait Islander people in the development of our recommendations. Arguably, despite the unbalanced spread of consumer advisors, it is still favourable to have members of the youth community contribute to this study rather than none. To mitigate this in future investigations or recommendations, the opportunity to refine and validate these findings with a larger, more diverse group is suggested.

It is to be stressed that the broad recommendations that resulted from this study were developed from a specific group of Australian young people; both within our focus groups and our Youth Advisory Group. The recommendations thus may not reflect the ideas and experiences of all Australian adolescents. The recommendations are suggested to be explored in multiple Australian educational settings for further refinement, in their path to implementation within national and state curricula. Promisingly, this study aligns with other research exploring Australia’s adolescent experience within sexuality education, providing more evidence toward the recommendations we developed.

## Conclusion

Australia’s current sexuality education needs reform and refinement to ensure it is relevant to the wants and needs of its current youth population. This project aimed to provide insight into perceptions of youth about their sexuality education and to co-design recommendations to improve sexuality education. The study revealed that youth consider the prevailing attitudes towards sex, the quality and content of sexuality education offered at schools, and the influence of external sources like social media, the internet and peers as significant issues in their school-based education. Despite these themes capturing primarily negative experiences, it introduced direction into advancements. Through co-designing recommendations with the Youth Advisory Group, we were able to make relevant, unambiguous recommendations to refine and contextualise current sexuality education practice. This study highlights the complex dynamics influencing sexuality education in an Australian context. This contributes to an increasingly comprehensive understanding of these dynamics, essential to improve national sexual and reproductive health outcomes.

## Data Availability

The datasets generated and/or analysed during the current study are not publicly available due to identifiable participant data but are available from J.S on reasonable request.

## Appendix 1

### Focus group discussion guide

Welcome and thank you for taking part in this focus group. We wanted to talk to you after the survey about some of the themes because your opinion is important for making decisions about the way young people are taught.

Introduction: This focus group discussion is designed to understand your current thoughts and feelings about the way you find and understand information about sexual and reproductive health. We want to know what is important to you, to help us with teaching students of the future about these topics. The focus group discussion will go up to ninety minutes but could be less. We are going to record this session so I can write everything that has been said today.

Anonymity: Despite being recorded, I can assure you that the discussion will be confidential and private. The tapes will be kept safely in a locked facility until they are transcribed, which means they will be written up, word for word, then they will be destroyed. The transcribed notes of the focus group will contain no information that would allow individual subjects to be linked to specific statements. No names, places, ages or other things will be included. The same is true for any reports we may write with the data. You should try to answer and comment as accurately and truthfully as possible. I and the other focus group participants would appreciate it if you would not discuss any comments, including those of other group members, outside of the focus group. If there are any questions or discussions that you do not wish to answer or participate in, you do not have to; however please try to answer and be as involved as possible.

Ground rules

- The most important rule is that only one person speaks at a time. There may be a temptation to jump in when someone is talking but please wait until they have finished.
- There are no right and wrong opinions, only difference between opinions
- You do not have to speak in any particular order
- When you do have something to say, please do so. There are many of you in the group and it is important that I hear from everyone
- You do not have to agree with the views of other people in the group
- You must respect the confidentiality of the group and keep what we discuss private
- You can leave this group at any time if you wish – or you can private message me in the chat if you are feeling uncomfortable and myself or [other researcher] will create a breakout room to chat with you
- Does anyone have any questions? (answers).
- OK, let’s begin

Warm up

- First, I’d like everyone to introduce themselves. Can you tell us your first name, and your favourite subject at school? (Research team to start)

Introductory question(s)

- I am just going to give you a couple of minutes to think about your experience of the survey. Is anyone happy to share their thoughts on what they thought of it?
  - ◦ Was it what you expected, did anything surprise you?

### Guiding questions (with prompts below each point)

- Do you think this survey will help us discover what young people should be taught about reproductive health and fertility?
  - ◦ Did you feel anything was missing from the survey?
- Have you ever thought about being a parent before?
  - ◦ Changed mind?
  - ◦ What went into those choices?
- What are the different sources you have used to get information about sexual and reproductive health?
  - ◦ Why did you choose this source(s)?
  - ◦ Which one was best? Why?
  - ◦ How do you know the information you are getting is reliable and true?
  - ◦ What makes it hard to find information you want?
  - ◦ What about X method makes it best for you?
- Onto the questions we asked you in the survey about reproductive and sexual health now. We sent you some information about the answers before this session today. Did any surprise you?
  - ◦ Why did that answer surprise you? Can you share what you originally thought?
  - ◦ Why didn’t that surprise you, can you tell me how you knew?
  - ◦ (Note: can elaborate on what is known/correct for each topic to inform young people)
- In these questions, you may have noticed we sometimes discussed averages, or the most common ways our bodies work. Why did you think we did this?
  - ◦ Do learning about what is common in reproductive function help you? Can you elaborate?
- We asked you about infertility too, have you thought or heard about how common it is for people to have trouble having children?
  - ◦ Do you think infertility is discussed as much as other sexual health topics like contraceptives or pregnancy?
  - ◦ How do you think people with infertility feel going through these struggles?
  - ◦ Do you think people are taught about infertility or fertility? When? How?
- Some of the survey was about anatomy, things like pregnancy, hormones, and fertilisation. Were these familiar to you, have you learned about it before?
  - ◦ How would you rate your knowledge of the reproductive system of the opposite sex?
  - ◦ Do you think anatomy relates to infertility? How?
- What are some other aspects of sexual and reproductive health that are important to you in your life right now?
  - ◦ Who would you ask for help in these issues? Where you would find information?
  - ◦ How would you know that’s the right information?
  - ◦ How can we help you find the right information?

Concluding question

- Of all the things we’ve discussed today, what would you say are the most important issues you would like other young people to know about reproductive health?

Conclusion

- Thank you for participating. This has been a very successful discussion
- Your opinions will be a valuable to our work
- We hope you have found the discussion interesting
- If there is anything you are unhappy with or wish to discuss, you can email me
- I would like to remind you that any comments featuring in this research will be anonymous

Student support prompts for facilitators

It is possible that a student may become upset during the focus group. It is important that they be removed from the group discussion by either leaving the Zoom or going into a breakout room with one of the researchers. It is important to acknowledge the emotion and express empathy rather than ignoring the emotion, or moving straight into problem solving, giving advice or making suggestions.

Acknowledgement and empathy simply means a short sentence naming the possible emotion, determining whether it is true and saying that we care about their struggle. For example:

- I can see you are feeling really frustrated right now. I’m sorry you are feeling so bad.
- I could be wrong but you seem a little worried. That must feel tough.
- I’m sorry you are feeling upset about that. It’s not fun to feel overwhelmed like that.
- I think I would also feel very sad if that was me.
- I’m sorry you are feeling so sad.

This helps the young person realise you care about their wellbeing and feelings are validated. You may then direct them toward support services either within the school (councillor, call parents), or the below services:

- Kids helpline 24 h nationwide phone line: ph 1800 55 1800
- Headspace youth mental health network: https://headspace.org.au/
- Lifeline Australia mental health phone line: ph 13 11 14
- Reach out youth mental health service: https://au.reachout.com
- 1800 respect sexual assault/domestic violence support 24 h: ph 1800 737 732
- Family Planning NSW sexual health provider: talkline 1300 658 886 or https://www.fpnsw.org.au/health-information/individuals/under-25s

## Appendix 2

**Table 4:** Key findings and supporting verbatim

Theme	Subtheme	Supporting verbatim
Culture of sex	Sex being taboo	“I think we copy like adults and what they see is like acceptable and what they think is like okay to talk about and so like from that young age you see like your teachers don't want to talk about it. Your parents don't want to talk about it, people on the internet that you found on YouTube are a little bit awkward about talking about it too. Um it kind of just gives this idea that like it's not something that you should talk about” (Female, 17, Bisexual)
		“Infections and diseases they’re definitely like this massive taboo like if you have one just don't say anything, and don't talk about them and they don't exist” (Female, 17, Bisexual)
	General poor sex culture	“So I'm gonna have sex, don't care about the consequences because in the end, If I knock someone up I can leave. That that's their mentality. It's a lot of peer pressure, it's kind of that societal expectation to, again, guy sleeps with girl, guy gets, you know, a high five from his friends. Girls sleeps with guy, girl gets slut shamed” (Female, 17)
		“As boys we have a tendency to have, to be honest, a sort of misogynistic, very old, poor, I don’t know, behaviour” (Male, 15, Heterosexual)
Content of sexuality education	Motives of sex for youth	“But I also think sex education really only teaches people what having sex is not how to have sex, because I feel like really just saying like a penis can go in a vagina that's, that's not the only way to have sex and also how to have pleasurable sex like not for reproduction. I feel like it's something that's not talked about, which people who are younger, are probably not doing it for reproduction” (Nonbinary, 17)
		“The whole idea of sex in terms of sex ed is for procreation rather than anyone's pleasure and being intimate with a partner, and the positives of that can generate, that idea—definitely not touched on” (Female, 17, Bisexual)
	Lack of fertility content	“I have never been taught anything to do with fertility, because if they are to teach anything in sex ed it’s more just trying to scare us into not doing it since it is basically not teaching us anything about fertility because that will kind of like, they think it's encouraging us” (Female, 15, Bisexual)
		“Yeah, we never touched on infertility at all or anything in regards to that which is something that I worry about personally like going into like my adult life” (Female, 17, Bisexual)
	Lack of LGBTQIA + inclusive content	“Our male teacher didn't know pretty much anything about the female system or how to have like sex with it and so when I asked how two women could do it or how two men, he had no idea” (Nonbinary, 17)
		“We basically just got taught the examples of sex basically we only got taught just you know just, male and female. And then contraception just, yeah just, you got a male, female, like, um, yeah I didn't know about that whole, you know, how you’d have to use it for two men” (Male, 18, Heterosexual)
	Sexually transmitted infection content	“I know what all the STIs are? Goodness no, like, do I understand them all? No, of course not. And the fact that they spread over so many, but didn't go into them in as much detail” (Female, 16, Bisexual)
		“Chlamydia. of course, I know what I know what the word is, I don't really know what it is. I know it's an STI. Right?” (Female, 17)
	Vague and inexplicit content	“I'm sure you've heard of it, the tea video for consent. And I feel like it's kind of it's kind of backwards, it defeats the purpose in my opinion because we have to be explicit to be educated.” (Female, 16, Heterosexual)
		“The tea video like people don't want an analogy of like don't force it down to someone’s throat. No don't force somebody to have sex if they don't want to wait. We're like unintentionally glossing things over and in that sense we’re lessening the idea of the consequence, like, okay, forcing tea down someone's throat you're going to burn your throat but or forcing someone to have sex you could give them an STI. You couldn't leave them with permanent trauma, you can leave them with a child.” (Female, 17)
Sources of sexual health information beyond formal education	Impact of digital media on sexuality understanding	“So I think a lot of the media like that, whether it be made movies, TV, pornography, whatever. A lot of the time they glorify and romanticise borderline assault and abuse and when we don't have adequate consent education within schools already, we can't see that when we're consuming it we consume it and idolise it” (Female, 17)
		“Obviously the media is a big portrayal even in movies that's where you first come across a lot of like interactions. And then the idea of consent is obviously not the core of a lot of these movies” (Female, 17, Bisexual)
		“And then people get very misinformed ideas when they consuming pornography and the way that relationships should work in terms of that I've seen people's ideas and things like that, and how skewed they are from reality type of thing, because of that, just not being able to separate that and, like, that's fiction, from like the real world type of thing” (Female, 17, Bisexual)
	Sexual health misinformation	“Yeah, and the internet is also a very dangerous place in terms of talking about sexual stuff, because you don't know who the age of the person is who you're talking to, the validity of the article you're looking at. Even if the source, you have is serious or satire, you never know, like, it's a complete grey area” (Agender, 17, Bisexual)
